# Multiorgan ultrastructural changes in rats induced in synthetic torpor

**DOI:** 10.3389/fphys.2024.1451644

**Published:** 2024-11-19

**Authors:** Sara Salucci, Timna Hitrec, Emiliana Piscitiello, Alessandra Occhinegro, Luca Alberti, Ludovico Taddei, Sabrina Burattini, Marco Luppi, Domenico Tupone, Roberto Amici, Irene Faenza, Matteo Cerri

**Affiliations:** ^1^ Department of Biomedical and Neuromotor Sciences – University of Bologna, Bologna, Italy; ^2^ Department of Biomolecular Sciences, Carlo Bo Urbino University, Urbino, Italy

**Keywords:** torpor, synthetic torpor, electron microscopy, hypothermia, raphe pallidus, liver, skeletal muscle, glycogen stores

## Abstract

Torpor is a state used by several mammals to survive harsh winters and avoid predation, characterized by a drastic reduction in metabolic rate followed by a decrease in body temperature, heart rate, and many physiological variables. During torpor, all organs and systems must adapt to the new low-energy expenditure conditions to preserve physiological homeostasis. These adaptations may be exploited in a translational perspective in several fields. Recently, many features of torpor were shown to be mimicked in non-hibernators by the inhibition of neurons within the brainstem region of the Raphe Pallidus. The physiological resemblance of this artificial state, called synthetic torpor, with natural torpor has so far been described only in physiological terms, but no data have been shown regarding the induced morphological changes. Here, we show the first description of the ultrastructural changes in the liver, kidney, lung, skeletal muscle, and testis induced by a 6-hours inhibition of Raphe Pallidus neurons in a non-hibernating species, the rat.

## Introduction

Torpor is a state characterized by a significant and reversible reduction in metabolic rate, accompanied by a corresponding decrease in numerous physiological parameters, including body temperature, heart rate, respiratory rate, cortical electrical activity, and generally, all bodily functions (Geiser, 2013; Heldmaier, et al., 2004; Sonntag and Arendt, 2019). It is a strategy employed by many mammals, such as the dormouse, bear, squirrel, hamster, bat, and others, to endure periods of resource scarcity or to diminish the risk of predation (Geiser, 2013; Ruf and Bieber, 2023).

During natural torpor, all organs and systems adjust to the condition of hypometabolism to ensure the maintenance of body homeostasis. These systemic adaptations, which involve changes in cellular biology and structure, are critical for the animal’s survival during the hypometabolic period (Boyer and Barnes, 1999; Giroud et al., 2020; Klug and Brigham, 2015). Generally, changes in cellular homeostasis can be reflected by changes in the ultrastructure of various cellular domains. In fact, a diverse array of ultrastructural adaptations has been observed in many organs during torpor (Boyer and Barnes, 1999; Brustovetsky et al., 1993; Burlington et al., 1972; Giroud et al., 2020; Klug and Brigham, 2015; Zancanaro et al., 2000) that may be at the base of the cellular protection commonly attributed to the hibernating phenotype (Giroud et al., 2020) and that a recent hypothesis attributes to ferroptosis-resistance (Sone and Yamaguchi, 2024).

Over the last decade, several procedures have been developed to simulate aspects of torpor in non-hibernating animals (Cerri et al., 2013; Takahashi et al., 2020; Tupone et al., 2013; Yang et al., 2023; Zakharova et al., 2019). Specifically, the inhibition of neurons in the Raphe Pallidus, a crucial thermoregulatory nucleus in the brainstem (Morrison and Nakamura, 2019), causes a considerable reduction in body temperature (Zaretsky et al., 2003), and has been shown to induce many physiological changes also observed in torpor (Cerri et al., 2013; Hitrec et al., 2021; Sgarbi et al., 2022). This induced state has been proposed to be called Synthetic Torpor (STor) (Cerri, 2017).

It is currently unclear how much STor replicates natural torpor in terms of physiology and ultrastructural anatomy. Therefore, to assess the degree of resemblance of STor to natural torpor more accurately, at least within the first few hours of hypothermia, we utilized light (LM) and transmission electron microscopy (TEM) to evaluate the ultrastructural status of key organs such as the liver, kidney, lung, skeletal muscle, and testis.

Moreover, this research is advancing our understanding of the translational potential of STor technology, which could significantly impact medicine and, in a more distant future, the field of space exploration (Cerri et al., 2016; Chouker et al., 2021; Puspitasari et al., 2021; Puspitasari et al., 2022).

## Methods

### Animals

Experiments were performed on 6 male Sprague-Dawley rats (250–300 g; Charles River). Upon arrival, the animals were acclimated for 1 week to standard laboratory conditions: a 12-hour light-dark (LD) cycle (lights on at 09:00 h and off at 21:00 h) with free access to food (4RF21 diet, Mucedola) and water, maintained at an ambient temperature (Ta) of 24.0°C ± 1.0°C. During the adaptation period, rats were pair-housed in Plexiglas cages (Techniplast) filled with dust-free wood shavings, and the bedding was replaced every 2 days. All the experiments were performed in compliance with DL 26/2014 and European Union Directive 2010/63/EU, under the oversight of the Central Veterinary Service of the University of Bologna, with the approval of the Italian National Health Authority (decree n° 262/2020-PR).

### Surgery

**FIGURE 1:**
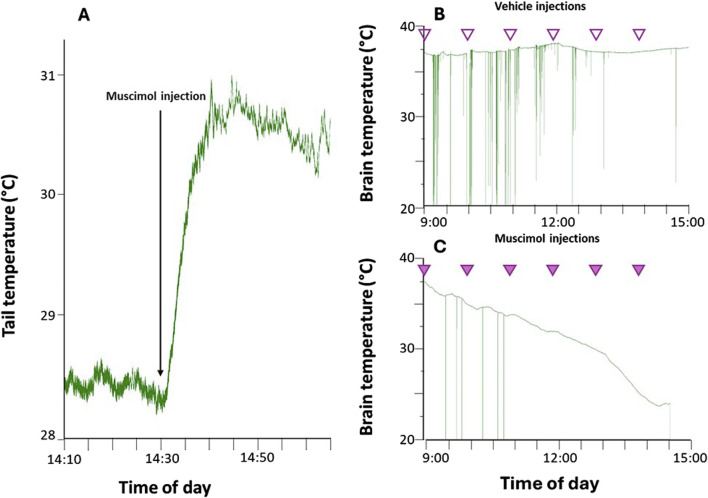
Representative examples of: panel **(A)** increase of tail temperature caused by the functional test used in an anaesthetized rat, after successful targeting of the region of the Raphe Pallidus (RPa) by muscimol nano-injection; panel **(B)** deep brain temperature recorded during repeated (1/h) Raphe Pallidus vehicle injection (empty arrowheads) in free-behaving rats; panel **(C)** deep brain temperature recorded during repeated (1/h) muscimol (1 mM, 100 nL) RPa injection (filled arrowheads) in free behaving rats. The green vertical lines indicate artifacts caused by the animal’s movement, which can occasionally disrupt the transmission of the temperature signal.

After 1 week of adaptation, rats underwent surgery under general anesthesia (Diazepam, 5 mg/kg i.m.; Ketamine-HCl, Imalgene 1,000, Merial, 100 mg/kg, i.p.), as previously described (Cerri et al., 2013). The following probes were stereotactically implanted: a thermistor (Thermometrics Corporation) placed in the right anterior hypothalamus to record the deep brain temperature (Tb) and a microinjection guide cannula (C315G-SPC; Plastics One; internal cannula extension below guide: +3.5 mm) targeting the Raphe Pallidus (RPa), coordinates (mm) −3.4 posterior from the interaural, 0.0 Lateral, −9.5 from the brain surface (Paxinos and Watson, 2007). Since the inhibition of RPa neurons induces vasodilation (Blessing and Nalivaiko, 2001; Cerri et al., 2010), the positioning of the guide cannula was considered correct if an increase in tail surface temperature was observed within 5 min following the injection of the GABA-A agonist muscimol (1mM, 100 nL) (Figure 1A). Subsequently, the entire surgical area, including the implanted probes and four stainless steel screws, was secured with dental resin (ResPal, Salmoiraghi Produzione Dentaria). Rats were treated with antibiotics (benzathine benzylpenicillin, 12.500.000 U.I., dihydrostreptomycin sulphate 5 g/100 mL, Rubrocillina Veterinaria, intramuscular, Intervet—1 mL/kg), analgesics (Carprofen—Rimadyl, Pfizer, subcutaneous—5 mg/kg) and rehydrated with 5 mL saline subcutaneously. After the surgery and throughout the whole experiment, the animals’ pain, distress or suffering were constantly evaluated using the Humane End Point (HEP) criteria. After 1 week of recovery under standard laboratory conditions, rats were moved to the experimental cages, placed in a thermoregulated and sound-attenuated box, and adapted to constant darkness and low Ta (15°C ± 1.0°C) for 48 hours. Tb signal was recorded following already published amplification, filtering, and digitalization parameters (Cerri et al., 2013).

### Experimental plan

On the experimental day, the animals were randomly assigned to one of the following two groups:

- Synthetic torpor group (STor, n = 3) received multiple microinjections (1 injection/hour, starting at 9 a.m.) of GABA-A agonist muscimol (1mM, 100 nL) within the RPa and successfully became hypothermic, reaching 24°C–22°C of brain temperature after 6 h from the first injection (Figure 1C).
- Normothermic group (Norm, n = 3) received multiple microinjections (1 injection/hour, starting at 9 a.m.) of artificial cerebrospinal fluid (aCSF, 100 nL; EcoCyte Bioscience) within the RPa (Figure 1B).

After 6 hours from the first microinjection, hypothermic and normothermic rats were euthanized by anaesthetic overdose (isoflurane 5%) and fresh tissue samples from liver, kidney, lung, skeletal muscle (quadriceps femoris) and testis were collected.

### Light and transmission electron microscopy

Samples were processed following previously published work (Curzi et al., 2012). Briefly, after rinsing the tissue in sodium phosphate buffer to remove excess blood, samples were fixed by immersion in 4% paraformaldehyde +1% glutaraldehyde in 0.1 M phosphate buffer (pH 7.4) for 24 h. Skeletal muscles were maintained under tension with pins and immediately fixed with 2.5% glutaraldehyde in a 0.1 M phosphate buffer for 3–6 h. All tissues were quickly minced into smaller (<1 mm^3^) fragments, postfixed in OsO_4_ (1% in PB), dehydrated with alcohol and embedded in araldite. Semithin sections, stained with 1% toluidine blue in distilled water at 60°C, were observed by means of a light microscope. Thin sections stained with uranyless and lead citrate were observed with a transmission electron microscope.

### Data analysis

Data analysis was carried out by two independent blinded analysts, and their results were averaged. To qualitatively compare Norm vs. STor and detect the main cellular differences in term of mitochondria, lipid droplets, glycogen granules and macrophage number, a total area of 1,000 μm^2^ has been considered.

Quantitative morphometric analysis was conducted on the same tissue sections used for morphological observations. Lipid droplets were counted within a total area of 10,000 μm^2^ using light microscopy images from semithin sections, analyzed for three rats under both experimental conditions. For mitochondria and glycogen aggregate area quantification, TEM images (1,000 μm^2^ total area) from ultrathin sections mounted on nickel grids (3 mm in diameter, 400 mesh) were used.

The total area of each tissue image, along with the areas occupied by lipid droplets, glycogen granules, and mitochondria, was measured using ImageJ 1.54j software (National Institutes of Health). Data were expressed as mean ± standard deviation. Graphs were generated using GraphPad 9, and statistical analysis was performed with an unpaired *t*-test, except for the Mitochondria area quantification, that was evaluated with the Mann-Whitney test, since the Shapiro-Wilk normality test was significant for this comparison. A p-value <0.05 was considered as statistically significant for all comparisons.

## Results

Morphological qualitative results from all organs have been summarized in Table 1.

**TABLE 1:** Shows an overview of the more relevant ultrastructural changes observed in synthetic torpor.

*Organ*	*Parameter*	*Norm*	*STor*
Liver	*glycogen droplets*	+	++++
	*lipid droplets*	++++	+
	*elongated and sprouting vessels*	+/−	+++
Kidney	No differences		
Lungs	*alveolar macrophages*	+++	+
Skeletal Muscle	*mitochondria and glycogen droplets*	++++	+
Testis	*lipid droplets in spermatogenic cells*	+	+++

### Liver

**FIGURE 2:**
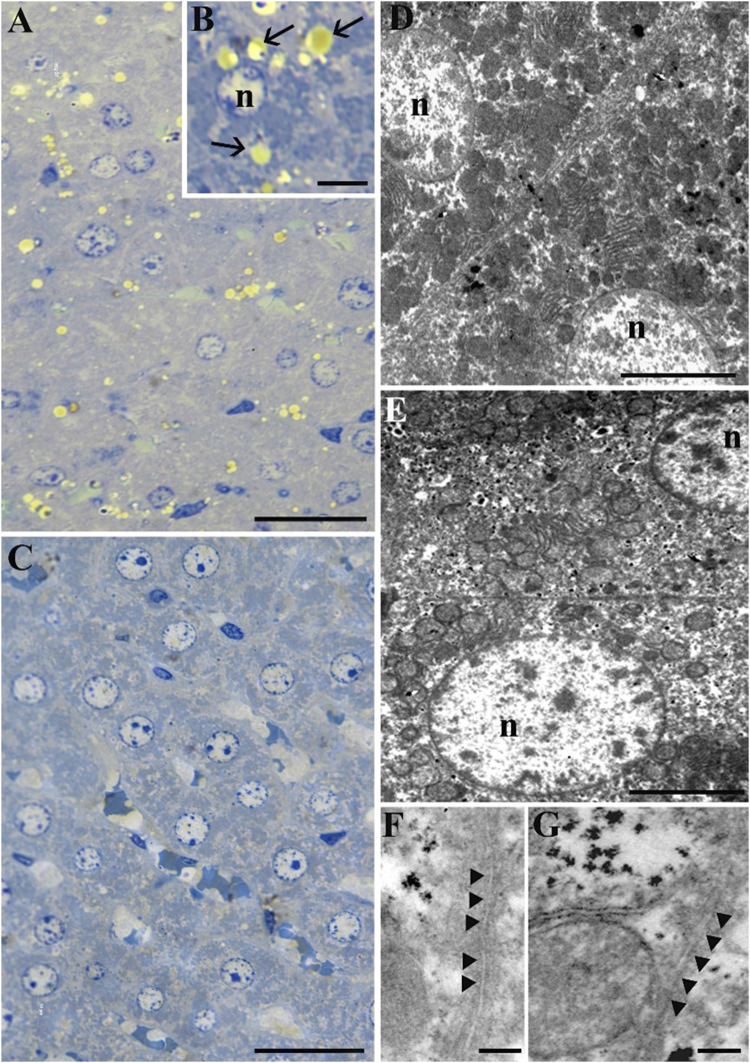
Light Microscopy **(A–C)** and Transmission Electron Microscopy **(D–G)** micrographs of samples from Norm **(A, B, D and F)** and STor **(C, E and G)** liver tissues. Scale bars: 20 µm for **(A, C)**; 5 µm for **(B, D and E)**; 200 nm for **(F, G)**. Black arrows and arrowheads indicate lipid droplets and the macula adherens junction, respectively. n: nucleus.

**FIGURE 3:**
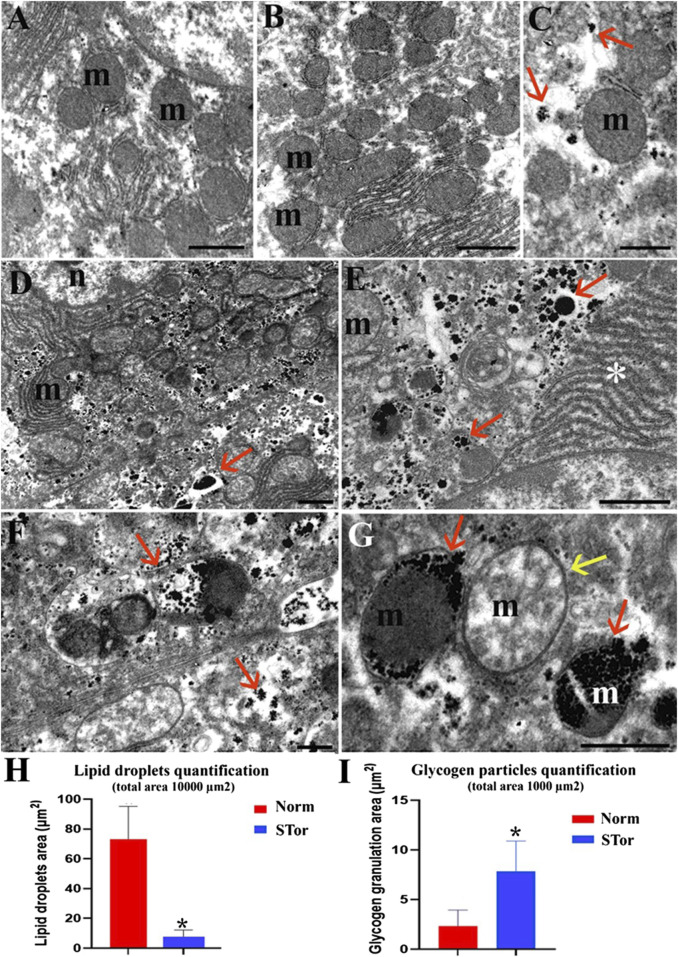
Transmission Electron Microscopy micrographs of Norm **(A–C)** and STor **(D–G)** liver tissues. High glycogen amount (arrows) in STor group **(D–G)** can be observed scattered in the cytoplasm **(D, E)** or accumulated in mitochondria **(F, G)**. Scale bars: 1 µm for **(A, B, D and E)**; 500 nm for **(C, F and G)** n: nucleus; m: mitochondria; asterisk: endoplasmic reticulum. **(H, I)** show quantification of lipid droplets and glycogen granulation areas. Scale bars: 1 μm for **(A, B, D and E)**; 500 nm for **(C, F and G)** n: nucleus; m: mitochondria; asterisk: endoplasmic reticulum; red arrows: glycogen granulation; yellow arrow: swollen mitochondrion. * = *p* < 0.05.

Morphological analysis of rat liver tissue has revealed a preserved structure of hepatic lobules in both experimental conditions (Figures 2A–C). As shown in Figure 2, the STor group (Figure 2C) exhibited a lower quantity of lipid droplets (black arrows) compared to the Norm group (Figures 2A, B). Morphometric analysis confirmed this result, since the area covered by lipid droplets is significantly (*p* = 0.007, t = 5.092, df = 4) lower in STor compared to Norm (Figure 3H). In the hepatocytes of both experimental groups, ultrastructural analysis revealed a dense cytoplasm with a centrally located nucleus (n), numerous mitochondria, and abundant endoplasmic reticulum (Figures 2D, E). The spacing between the plasma membranes at the macula adherens junction level appears larger in STor (approximately 42.2 nm, Figure 2G) compared to Norm (approximately 19.8 nm, Figure 2F).

Notably, at higher magnification, the STor group (Figures 3D, E) showed a greater number of glycogen granules (arrows) compared to the Norm group (Figures 3A–C). These granules were dispersed throughout the cytoplasm, accumulated in degradative vacuoles, or localized within mitochondria (Figures 3F, G). In fact, the quantification of the area covered by glycogen granulation is significantly (*p* = 0.049, t = 2.796, df = 4) higher in Stor compared to Norm (Figure 3I).

Occasionally, some mitochondria in the STor group appeared swollen (yellow arrow) and lost the regular distribution of mitochondrial cristae (Figures 3F, G).

**FIGURE 4:**
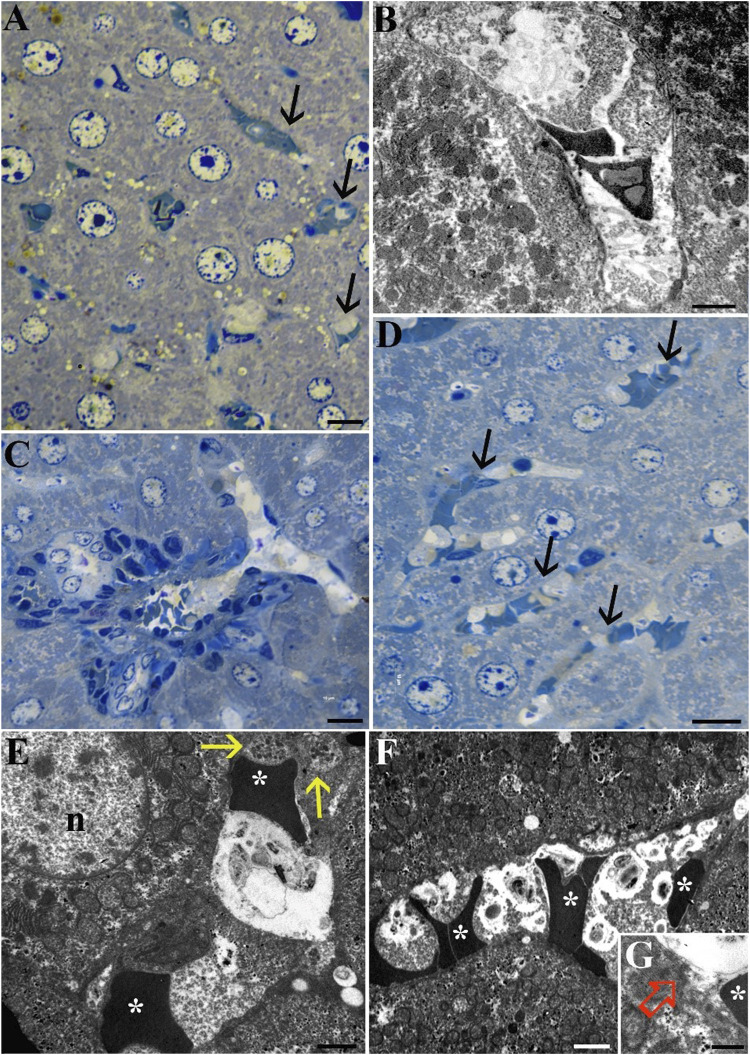
Light Microscopy **(A, C and D)** and Transmission Electron Microscopy **(B, E, F and G)** micrographs of Norm **(A, B)** and STor **(C, D, E, F and G)** liver tissues. The liver sinusoidal capillaries (black arrows) of STor group show angiogenic sprouts **(C, D)**. At the utrastructural level, erythrocytes (asterisks) and platelets (yellow arrows) are observed **(E, F)** as well as the preserved sinusoidal endothelium (G, empty red arrow). Scale bars: 10 µm for **(A, C, D)**; 2 µm for **(B, E, F)**; 0.5 µm for **(G)** n: nucleus.

Furthermore, while sinusoidal capillaries (arrows) were uniformly distributed throughout the liver parenchyma of the Norm group (Figures 4A, B), those in the STor group were characterized by an elongated and denser distribution (Figures 4C–F). The presence of sprouting among these capillaries suggests the potential activation of angiogenesis in the liver samples from the STor group.

### Kidney

**FIGURE 5:**
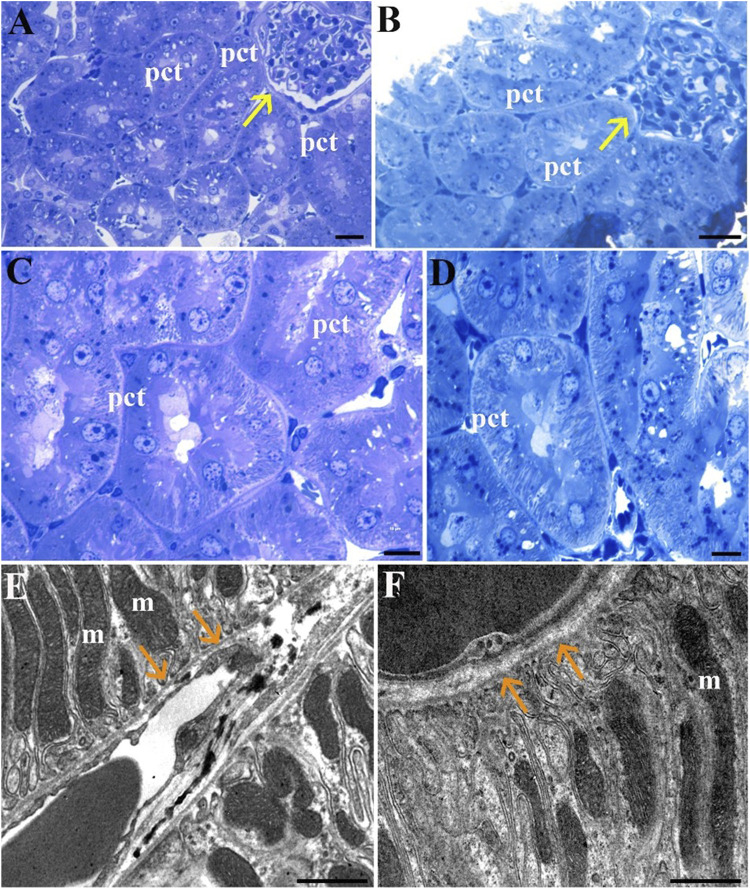
Light Microscopy **(A–D)** and Transmission Electron Microscopy **(E, F)** micrographs of Norm **(A, C and E)** and STor **(B, D and F)** kidney tissues. Proximal convoluted tubules (pct) appear around the renal corpuscles (yellow arrows) and show a preserved morphology in both experimental groups. Scale bars: 20 µm for **(A, B)**; 10 µm for **(C, D)**; 1 µm for **(E, F)**. Orange arrows: proximal convoluted tubules’ basement membrane, m: mitochondria.

In the kidney, LM reveals comparable morphology between the Normothermia (Norm) group (Figures 5A, C) and the STor group (Figures 5B, D). Transverse sections of the renal cortex display proximal convoluted tubules (pct) with their microvillar components, as well as Malpighian corpuscles (yellow arrows). Ultrastructurally, the proximal convoluted tubules’ thin basement membrane (Figures 5E, F; orange arrows) is shown to have basal infoldings and is populated with an abundance of elongated mitochondria (m) in both experimental groups.

**FIGURE 6:**
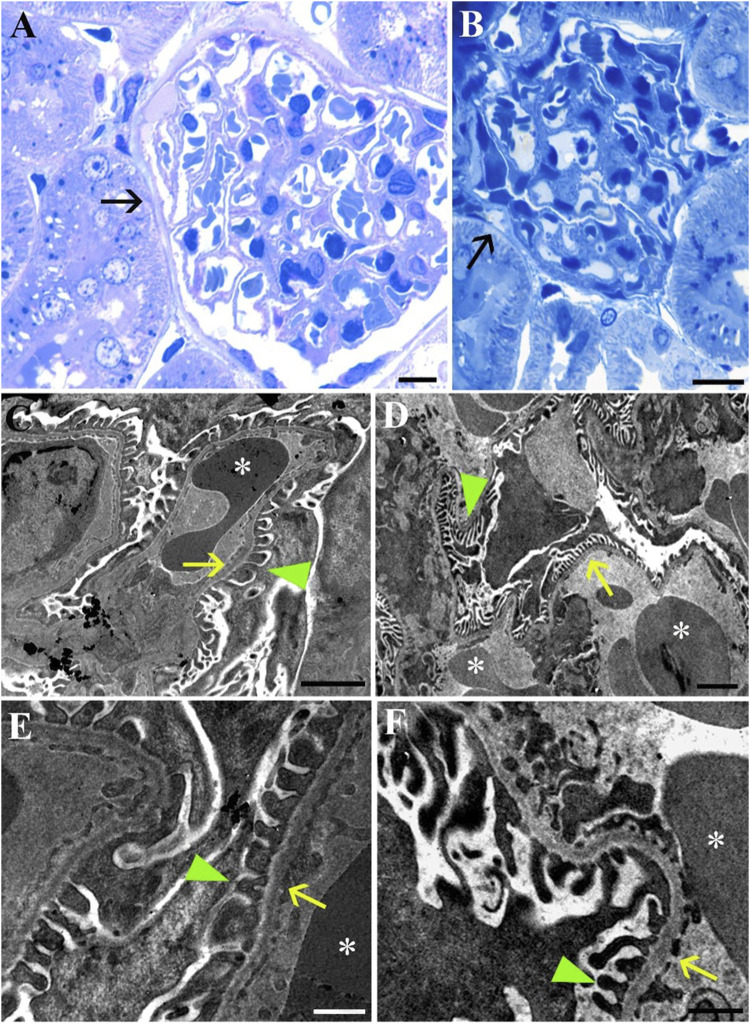
Light Microscopy **(A, B)** and Transmission Electron Microscopy **(C–F)** micrographs of Norm **(A, C and E)** and STor **(B, D and F)** kidney tissues. In both conditions Bowman’s capsule (black arrows), glomerular capillaries and podocyte foot processes (green arrowheads) that cover the glomerular basement membrane separating it from the fenestrated endothelium (yellow arrows) can be seen. *: erythrocytes. Scale bars: 10 µm for **(A, B)**; 2 µm for **(C, D)**, 500 nm for **(E, F)**.

LM imaging of the Bowman’s capsule and the glomerulus did not reveal any differences between the groups (Figures 6A, B). This finding is supported by the ultrastructural analysis, which provides detailed insights into the interactions between the glomerulus and the podocytes (Figures 6C, D). At higher magnifications, the foot processes of the podocytes (green arrowheads) are distinctly visible in both Norm (Figures 6C, E) and STor (Figures 6D, F) groups. These foot processes are separated by slit diaphragms and are anchored to the glomerular basement membrane. The glomerular basement membrane, acting as an interface between the podocyte foot processes and the fenestrated endothelial cells (yellow arrows) of the capillary loops, displays segments of erythrocytes (asterisk).

### Lungs

**FIGURE 7:**
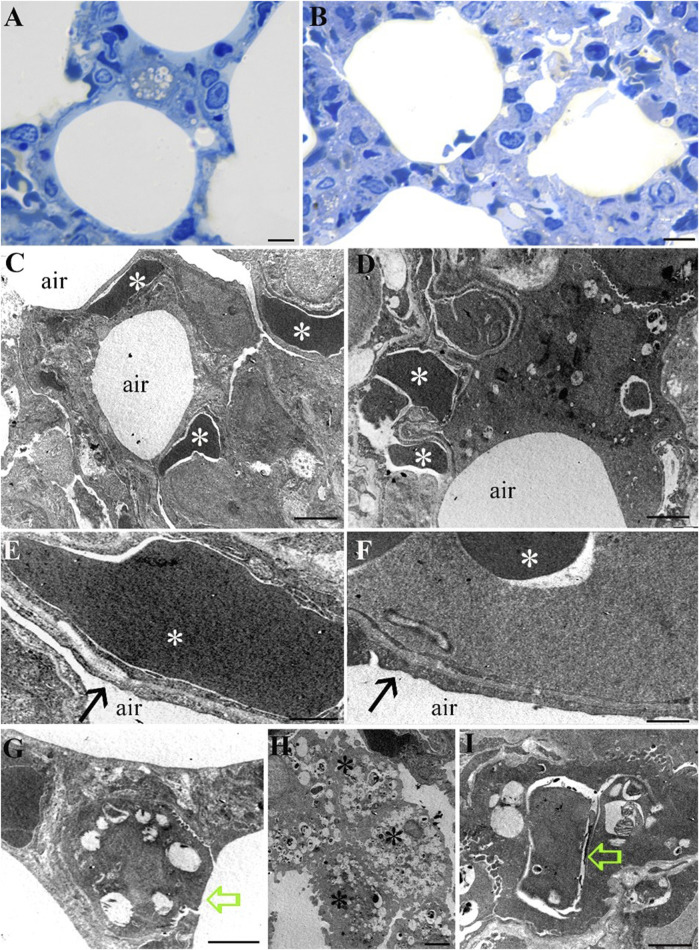
Light Microscopy **(A, B)** and Transmission Electron Microscopy **(C–I)** micrographs of Norm **(A, C, E, G and H)** and STor **(B, D, F and I)** lung tissues. Alveolar sacs show no differences between the two conditions **(A, B)**. No differences have also been observed at the ultrastructural level. Capillaries, containing red blood cells (white asterisks) are scattered into the lung parenchyma **(C, D)** and some of them contact type I pneumocytes (black arrows) to generate the air-blood membrane **(E, F)**. Type II pneumocytes **(G, I)** can be observed in both conditions (green empty arrows). Diffuse macrophages appear in Norm group (H, black asterisks). Scale bars: 10 µm for **(A, B)**; 2 µm for **(C, D, G and H)**; 500 nm for **(E, F)**; 1 µm for **(I)**.

Morphological analysis of lung tissue in both the Norm and STor groups has revealed preserved organization of the alveolar sacs both in LM (Figures 7A, B) and TEM observations (Figures 7C–I). The cellular components of the alveolar wall, including endothelial cells with red blood cells (asterisks), as well as pneumocytes type I, were evident in the ultrastructural images and exhibited no morphological differences between the Norm (Figures 7C, E) and STor groups (Figures 7D, F). At higher magnification, the blood-air barrier was discernible, as well as the interface between pericytes and extensions of pneumocyte type I (black arrows), which displayed similar morphology in both examined conditions (Figures 7E, F).

Pneumocytes type II (empty green arrows), characterized by their distinctive multilamellar bodies and a microvillar surface, appeared comparable in the Norm (Figure 7G) and STor (Figure 7I) groups. Notably, alveolar macrophages (black asterisks) were more abundant in the Norm samples (Figure 7H) compared to those from the STor group, suggesting a possible variation in immune cell distribution between the two states.

### Skeletal muscle

**FIGURE 8:**
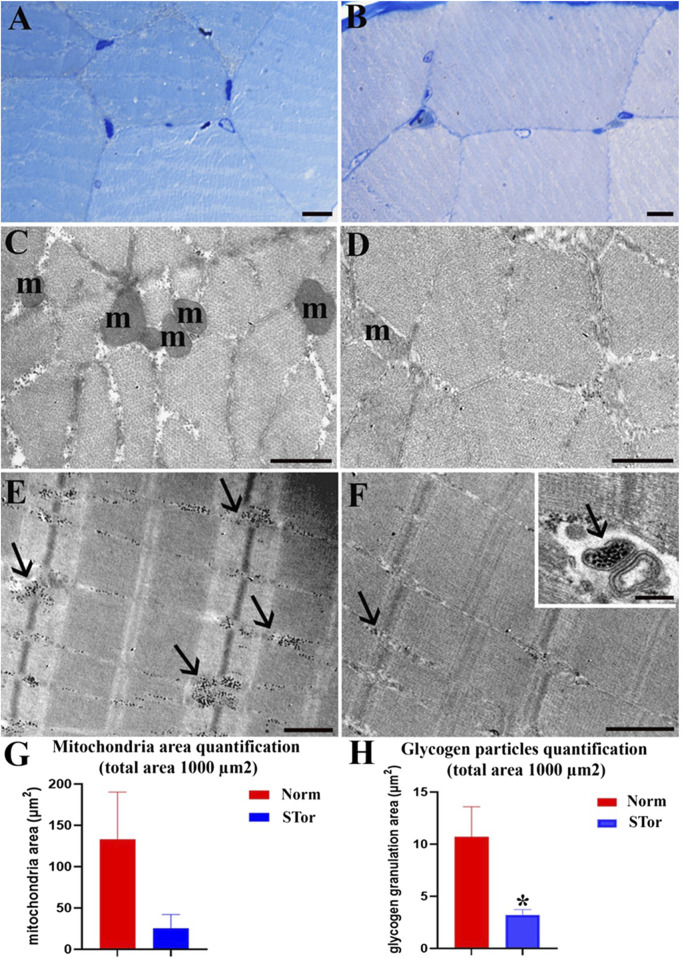
Light Microscopy **(A, B)** and Transmission Electron Microscopy **(C–F)** micrographs of Norm **(A, C and E)** and STor **(B, D, F)**, inset **(F)** skeletal muscle tissues. No differences between myofibers can be seen **(A, B)**. After ultrastructural analysis, a reduction in the number of mitochondria (m) and glycogen granules (arrows) can be observed in Stor **(D, F)**. In **(G, H)**, graphs show mitochondrial density quantification, and the area covered by glycogen granulation. Scale bars: 10 µm for **(A, B)**; 500 nm for **(C–F)**; 200 nm for inset **(F)**. * = *p* < 0.05.

Morphological evaluation of skeletal muscle tissue under LM reveals no discernible differences in muscle fiber organization between the Norm group (Figure 8A) and the STor group (Figure 8B). Muscle fibers maintain their physiological alignment and distribution in both states. At the ultrastructural level, however, some differences between the two experimental groups can be observed. The STor group (Figures 8D, F) exhibited a lower number of mitochondria (m) and glycogen granules (arrows) compared to the Norm group (Figures 8C, E), both in transverse (Figures 8C, D) and longitudinal (Figures 8E, F) sections. Occasionally, glycogen granules (arrow) are sequestered within vesicles, as noted in the insert of Figure 8F. Despite the qualitative results, quantitative analysis did not show a significant difference in mitochondria area quantification between groups (Figure 8G), whereas STor showed a significantly lower amount of glycogen particles compared to Norm (*p* = 0.0116, t = 4.407, df = 4, Figure 8H).

The structural integrity of the sarcomeres remained consistent under both experimental conditions. The sarcomeres, characterized by their well-organized thick and thin filaments, displayed a preserved structure, indicating that the essential muscle contraction mechanism remained unaltered during synthetic torpor (see Figures 8E, F). This preservation of sarcomere structure underscores the resilience of muscle architecture, even amidst changes in cellular components such as mitochondria and glycogen reserves in response to shifts in physiological states like hypothermia.

### Testis

**FIGURE 9:**
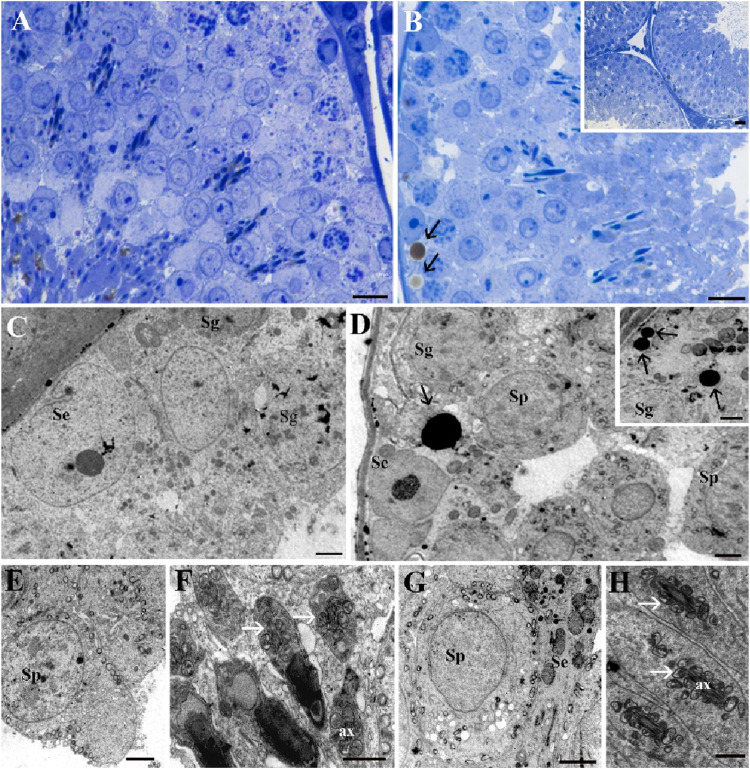
Light Microscopy **(A, B)** and Transmission Electron Microscopy **(C–G)** micrographs of Norm **(A, C, E and F)** and STor **(B)**, inset **(B, D)**, inset **(D, G and H)** testes. Seminiferous tubules and details of spermatogenic cells can be observed without significant differences between the experimental groups. Lipid droplets (black arrows) appear more abundant in the STor group **(B, D)** and inset **(D)**. In both samples, spermatocytes show a central nucleus and circular mitochondria localized around the plasma membrane **(E, G)**. In late stages of spermatogenesis **(F, H)**, round mitochondria (white arrows) surround the axoneme (ax). Scale bars: 10 µm for **(A, B)**; 20 µm for inset **(B)**; 2 µm for **(C–G)** and inset **(D)**; 1 µm for **(H)**. Se: Sertoli cells; Sg: spermatogonia; Sp: spermatocytes.

The testes of both the Norm group (Figures 9A, C, E, F) and the STor group (Figure 9B, inset B, D, inset D, G, H) rats displayed seminiferous tubules with a typical appearance, usually circular in cross-section (inset 9B) with regular contours. The tubules were populated with all stages of spermatogenic cells, and notably, lipid droplets (black arrows) were particularly prevalent in the STor samples (Figures 9B, D). The basal lamina surrounding the seminiferous tubules remained intact, with the interstitial tissue well preserved in both conditions.

In both groups, the organization of cellular types within the seminiferous tubules was preserved, with the nuclei of Sertoli cells (Se) and spermatogonia (Sg) being readily identifiable. Their subcellular organelles were well preserved, as shown in Figures 9C, D. The primary spermatocytes (Sp; Figures 9E, G) showed a central nucleus with mitochondria characteristically situated near the plasma membrane, in line with known structures.

According to Godet and colleagues (Godet et al., 2008), the progression of meiosis relies on the close contact between spermatocytes and Sertoli cells, and this vital interaction was maintained in the STor samples (Figure 9G). Additionally, transverse sections of late-stage spermatids in both control and treated conditions (Figures 9F, H) show mitochondria that are regularly arranged, forming a collar or sheath around the axonemal core, which indicates preserved spermiogenesis.

This thorough examination of the testicular architecture highlights the stability of the spermatogenic process and the integrity of the spermatocyte-Sertoli cell interaction, even in treated conditions that may emulate physiological changes such as those induced by environmental or experimental factors.

## Discussion

In this study, we present the first morphological analysis of organ adaptation during an induced artificial torpor state in rats. We observed distinct organ-specific changes; some are consistent with those noted in natural torpor, while others appear unique to the synthetic torpor condition.

The main finding we observed is a significant increase in glycogen droplets in the liver during synthetic torpor. The first interpretation of this observation revolves around the methods used to induce synthetic torpor, specifically through pharmacological inhibition of neurons within the rostral Raphe Pallidus (RPa), a key thermoregulatory region in the brain (Morrison and Nakamura, 2019). These neurons have multisynaptic connections through the sympathetic nervous system with various metabolically active organs, including brown adipose tissue and the tail (via VGLUT-3 positive neurons) (Cano et al., 2003; Nakamura et al., 2004; Nakamura et al., 2002), white adipose tissue (Nguyen et al., 2014), the thyroid (Kalsbeek et al., 2000), the heart (Standish et al., 1995; Ter Horst et al., 1993), the kidney (Huang and Weiss, 1999), the adrenergic cells in the adrenal gland (Morrison and Cao, 2000), the bones (Denes et al., 2005), the skeletal muscle (Billig et al., 1999), the pancreas (Streefland et al., 1998), and the liver (Kalsbeek et al., 2004). Activation of RPa neurons leads to an increase in metabolic rate and thermogenesis both in anaesthetized (Morrison et al., 1999) and awake (Cerri et al., 2010) rats. This suggests a potential regulatory role for the RPa-to-liver pathway in managing hepatic regulation of energy. Activation of these pathways may signal the liver to mobilize energy reserves to meet heightened metabolic demands, whereas inhibition might cause an accumulation of energy substrates, prompting their storage as glycogen droplets due to reduced thermogenic activity. However, it cannot be excluded that the increase in glycogen is not centrally driven, but is rather a consequence of the effect of hypothermia, that could directly stimulate hepatocytes to increase glycogen accumulation. At the same time, the decrease in hepatocytes lipid droplets points towards a shift in the metabolic substrate used from glucose to lipids, in accordance with the idea of a neural regulation over the liver regulation of metabolism. Distinguishing the effects mediated by neural pathways from those resulting from temperature changes requires further investigation.

Interestingly, we observed sprouting in liver capillaries, suggesting the potential activation of angiogenesis in the liver samples from the STor group. To the best of our knowledge there is no evidence about a possible role of RPa in promoting angiogenesis, nor has it been described in hibernators. Future studies could aim at understanding the functional role of this phenomenon.

Lastly, structural analysis revealed a less rigid macula adherens in the STor group compared to Norm. The macula adherens has been shown to have a dynamic nature (Takeichi, 2014) and has been suggested to have a role in regulation of bile release (Kojima et al., 2003) Therefore, it is possible that synthetic torpor may influence bile release by the liver, possibly due to a shift in energy substrate from carbohydrates to lipids. The functional significance of this modification is still to be explored.

Regarding the skeletal muscle, our observations in quadriceps femoris included a significant reduction in glycogen. Mitochondrial density appeared to be qualitatively lower in synthetic torpor, however, this impression was not confirmed by quantitative analysis, likely due to high variability in the number of mitochondria of the control group. The changes we observed could be part of the selective preservation of muscle function, which could be exploited for applications where preserving muscle strength during prolonged inactivity would be beneficial (Cerri et al., 2021; Chouker et al., 2019). This effect can also be mediated by the RPa neurons or be temperature dependent. Regarding ultrastructural morphology, little changes in muscle cells were observed. It still needs to be determined whether these observations apply to all muscle tissues or are specific to the one we examined.

Interestingly, lipid droplets increased in spermatogenic cells, possibly reflecting a pause in the energetically demanding process of spermatogenesis during the hypometabolic state induced by RPa inhibition. No significant structural changes were noted in the kidneys, and the lung tissue exhibited fundamental stability in alveolar architecture and cellular characteristics despite the physiological shifts.

Our observations are mostly consistent with what has been described for natural hibernators, although not much ultrastructural data is available and although the duration of the hypothermia/hypometabolism bout is substantially longer in hibernation. Moreover, the difference in body mass between large and small hibernators may be quite significant and could therefore induce species-specific changes in selective tissues.

Regarding the liver, our observations contrast with the changes in glycogen droplets in natural torpor (Adodina et al., 1987; Malatesta et al., 2002), but the changes in lipid droplets may be consistent with the change in substrates used described for hibernators (Carey et al., 2003; Heldmaier et al., 1999).

Regarding the kidney, we found the renal architecture was maintained during synthetic torpor, similarly to what has been described for natural hibernators. In the latter, the renal architecture remains well-preserved with only minor morphological changes reported, such as many basolateral elongated mitochondria, dilation of the endoplasmic reticulum, and an increased amount of lysosomes (Soria-Milla and Coca-Garcia, 1986; Zancanaro et al., 1999; Zimny and Levy, 1971).

Regarding the lungs, our findings indicate a preserved organization of alveolar sacs during synthetic torpor; however, we observed a decrease in alveolar macrophage distribution. This reduction may suggest immune adaptations similar to those that have been observed in natural hibernation (Bouma et al., 2010). Such similarities could highlight shared strategies for energy conservation during hypometabolic states, although this requires further investigations.

In general, it is known that during hibernation many changes occur in the skeletal muscle: mitochondrial metabolism (Brown et al., 2012) and ATP utilization are reduced (Chazarin et al., 2019), protein and oxidative energy metabolism are downregulated (Miyazaki et al., 2022), and profound myosin structure remodeling occurs (Lewis et al., 2024), highlighting the translational potential for this kind of adaptation (Stenvinkel et al., 2013). The reduction in glycogen and mitochondrial density in skeletal muscle we observed has already been described in larger hibernators such as the bear (Chazarin et al., 2019). However, contrasting evidence in smaller hibernators shows an increase in muscle glycogen and mitochondria during torpor (Wang et al., 2019), suggesting that this process is variable across species, possibly as a function of body size, and most likely varies depending on the type of muscle and fiber studied. Regarding ultrastructural morphology, little changes were also described in natural hibernators (Malatesta et al., 2020; Malatesta et al., 2009).

Regarding the testis, seasonal hibernators undergo a drastic reshaping of testis mass and structure, that is strongly influenced by the seasonality of torpor (Barnes et al., 1986). Compared to natural hibernators (Reznik-Schuller and Reznik, 1974), rats do not show a reduction in the number of spermatids. Among the many possible reasons to explain this, the shorter duration of STor compared to natural episodes of torpor may prevent this adaptation from manifesting.

In summary, our TEM analysis did not reveal significant alterations in cellular fine structure across the examined tissues, suggesting that the main morphological changes during synthetic torpor are associated with shifts in lipid metabolism. Moreover, the adaptations observed appear to resemble what has been described in instances of natural torpor, suggesting that the inhibition of RPa neurons may physiologically mimic some of the torpor-induced organ adaptations. RPa neurons were in fact suggested to be inhibited at torpor onset (Hitrec et al., 2019). This aligns with previous studies indicating that synthetic torpor, although induced with a different method, does not appear to significantly compromise cellular functions (Puspitasari et al., 2022).

We are aware that 6 h of synthetic torpor may not compare adequately with weeks of hibernation and that our procedure does not replicate all the features of natural torpor. Nevertheless, these insights into the cellular adaptations during artificial torpor provide a valuable foundation for understanding organ-specific responses. While the potential therapeutic implications for humans, particularly in clinical scenarios requiring metabolic modulation, are intriguing, they remain speculative at this stage and warrant further investigation.

## Data Availability

The raw data supporting the conclusions of this article will be made available by the authors, without undue reservation.

## References

[B1] Adodina L. V. Guvakova T. V. Filiushina E. E. Shmerling M. D. (1987). Ultrastructure of cells from the liver-endocrine pancreas system in hibernating animals. Arkh Anat. Gistol. Embriol 92, 78–86. 3300609

[B2] Barnes B. M. Kretzmann M. Licht P. Zucker I. (1986). The influence of hibernation on testis growth and spermatogenesis in the golden-mantled ground squirrel, spermophilus lateralis. Biol. Reprod. 35, 1289–1297. 10.1095/biolreprod35.5.1289 3828438

[B3] Billig I. Foris J. M. Card J. P. Yates B. J. (1999). Transneuronal tracing of neural pathways controlling an abdominal muscle, rectus abdominis, in the ferret. Brain Res. 820, 31–44. 10.1016/s0006-8993(98)01320-1 10023028

[B4] Blessing W. W. Nalivaiko E. (2001). Raphe magnus/pallidus neurons regulate tail but not mesenteric arterial blood flow in rats. Neuroscience 105, 923–929. 10.1016/s0306-4522(01)00251-2 11530230

[B5] Bouma H. R. Carey H. V. Kroese F. G. (2010). Hibernation: the immune system at rest? J. Leukoc. Biol. 88, 619–624. 10.1189/jlb.0310174 20519639

[B6] Boyer B. B. Barnes B. M. (1999). Molecular and metabolic aspects of mammalian hibernation: expression of the hibernation phenotype results from the coordinated regulation of multiple physiological and molecular events during preparation for and entry into torpor. BioScience 49, 713–724. 10.2307/1313595

[B7] Brown J. C. Chung D. J. Belgrave K. R. Staples J. F. (2012). Mitochondrial metabolic suppression and reactive oxygen species production in liver and skeletal muscle of hibernating thirteen-lined ground squirrels. Am. J. Physiol. Regul. Integr. Comp. Physiol. 302, R15–R28. 10.1152/ajpregu.00230.2011 21993528

[B8] Brustovetsky N. N. Egorova M. V. Iljasova E. N. Bakeeva L. E. (1993). Relationship between structure and function of liver mitochondria from hibernating and active ground squirrels, citellus undulatus. Comp. Biochem. Physiol. B 106, 125–130. 10.1016/0305-0491(93)90017-y 8403844

[B9] Burlington R. F. Bowers W. D. Jr. Daum R. C. Ashbaugh P. (1972). Ultrastructural changes in heart tissue during hibernation. Cryobiology 9, 224–228. 10.1016/0011-2240(72)90037-5 5045630

[B10] Cano G. Passerin A. M. Schiltz J. C. Card J. P. Morrison S. F. Sved A. F. (2003). Anatomical substrates for the central control of sympathetic outflow to interscapular adipose tissue during cold exposure. J. Comp. Neurol. 460, 303–326. 10.1002/cne.10643 12692852

[B11] Carey H. V. Andrews M. T. Martin S. L. (2003). Mammalian hibernation: cellular and molecular responses to depressed metabolism and low temperature. Physiol. Rev. 83, 1153–1181. 10.1152/physrev.00008.2003 14506303

[B12] Cerri M. (2017). The central control of energy expenditure: exploiting torpor for medical applications. Annu. Rev. Physiol. 79, 167–186. 10.1146/annurev-physiol-022516-034133 27813827

[B13] Cerri M. Hitrec T. Luppi M. Amici R. (2021). Be cool to be far: exploiting hibernation for space exploration. Neurosci. Biobehav Rev. 128, 218–232. 10.1016/j.neubiorev.2021.03.037 34144115

[B14] Cerri M. Mastrotto M. Tupone D. Martelli D. Luppi M. Perez E. (2013). The inhibition of neurons in the central nervous pathways for thermoregulatory cold defense induces a suspended animation state in the rat. J. Neurosci. 33, 2984–2993. 10.1523/JNEUROSCI.3596-12.2013 23407956 PMC6619194

[B15] Cerri M. Tinganelli W. Negrini M. Helm A. Scifoni E. Tommasino F. (2016). Hibernation for space travel: impact on radioprotection. Life Sci. Space Res. (Amst). 11, 1–9. 10.1016/j.lssr.2016.09.001 27993187

[B16] Cerri M. Zamboni G. Tupone D. Dentico D. Luppi M. Martelli D. (2010). Cutaneous vasodilation elicited by disinhibition of the caudal portion of the rostral ventromedial medulla of the free-behaving rat. Neuroscience 165, 984–995. 10.1016/j.neuroscience.2009.10.068 19895871

[B17] Chazarin B. Storey K. B. Ziemianin A. Chanon S. Plumel M. Chery I. (2019). Metabolic reprogramming involving glycolysis in the hibernating brown bear skeletal muscle. Front. Zool. 16, 12. 10.1186/s12983-019-0312-2 31080489 PMC6503430

[B18] Chouker A. Bereiter-Hahn J. Singer D. Heldmaier G. (2019). Hibernating astronauts-science or fiction? Pflugers Arch. 471, 819–828. 10.1007/s00424-018-2244-7 30569200 PMC6533228

[B19] Chouker A. Ngo-Anh T. J. Biesbroek R. Heldmaier G. Heppener M. Bereiter-Hahn J. (2021). European space agency's hibernation (torpor) strategy for deep space missions: linking biology to engineering. Neurosci. Biobehav Rev. 131, 618–626. 10.1016/j.neubiorev.2021.09.054 34606822

[B20] Curzi D. Salucci S. Marini M. Esposito F. Agnello L. Veicsteinas A. (2012). How physical exercise changes rat myotendinous junctions: an ultrastructural study. Eur. J. Histochem 56, e19. 10.4081/ejh.2012.19 22688300 PMC3428968

[B21] Denes A. Boldogkoi Z. Uhereczky G. Hornyak A. Rusvai M. Palkovits M. (2005). Central autonomic control of the bone marrow: multisynaptic tract tracing by recombinant pseudorabies virus. Neuroscience 134, 947–963. 10.1016/j.neuroscience.2005.03.060 15994021

[B22] Geiser F. (2013). Hibernation. Curr. Biol. 23, R188–R193. 10.1016/j.cub.2013.01.062 23473557

[B23] Giroud S. Habold C. Nespolo R. F. Mejias C. Terrien J. Logan S. M. (2020). The torpid state: recent advances in metabolic adaptations and protective mechanisms^†^ . Front. Physiol. 11, 623665. 10.3389/fphys.2020.623665 33551846 PMC7854925

[B24] Godet M. Sabido O. Gilleron J. Durand P. (2008). Meiotic progression of rat spermatocytes requires mitogen-activated protein kinases of sertoli cells and close contacts between the germ cells and the sertoli cells. Dev. Biol. 315, 173–188. 10.1016/j.ydbio.2007.12.019 18234180

[B25] Heldmaier G. Klingenspor M. Werneyer M. Lampi B. J. Brooks S. P. Storey K. B. (1999). Metabolic adjustments during daily torpor in the djungarian hamster. Am. J. Physiol. 276, E896–E906. 10.1152/ajpendo.1999.276.5.E896 10329984

[B26] Heldmaier G. Ortmann S. Elvert R. (2004). Natural hypometabolism during hibernation and daily torpor in mammals. Respir. Physiol. Neurobiol. 141, 317–329. 10.1016/j.resp.2004.03.014 15288602

[B27] Hitrec T. Luppi M. Bastianini S. Squarcio F. Berteotti C. Lo Martire V. (2019). Neural control of fasting-induced torpor in mice. Sci. Rep. 9, 15462. 10.1038/s41598-019-51841-2 31664081 PMC6820542

[B28] Hitrec T. Squarcio F. Cerri M. Martelli D. Occhinegro A. Piscitiello E. (2021). Reversible tau phosphorylation induced by synthetic torpor in the spinal cord of the rat. Front. Neuroanat. 15, 592288. 10.3389/fnana.2021.592288 33603651 PMC7884466

[B29] Huang J. Weiss M. L. (1999). Characterization of the central cell groups regulating the kidney in the rat. Brain Res. 845, 77–91. 10.1016/s0006-8993(99)01937-x 10529446

[B30] Kalsbeek A. Fliers E. Franke A. N. Wortel J. Buijs R. M. (2000). Functional connections between the suprachiasmatic nucleus and the thyroid gland as revealed by lesioning and viral tracing techniques in the rat. Endocrinology 141, 3832–3841. 10.1210/endo.141.10.7709 11014240

[B31] Kalsbeek A. La Fleur S. Van Heijningen C. Buijs R. M. (2004). Suprachiasmatic gabaergic inputs to the paraventricular nucleus control plasma glucose concentrations in the rat via sympathetic innervation of the liver. J. Neurosci. 24, 7604–7613. 10.1523/JNEUROSCI.5328-03.2004 15342726 PMC6729629

[B32] Klug B. J. Brigham R. M. (2015). Changes to metabolism and cell physiology that enable mammalian hibernation. Springer Sci. Rev. 3, 39–56. 10.1007/s40362-015-0030-x

[B33] Kojima T. Yamamoto T. Murata M. Chiba H. Kokai Y. Sawada N. (2003). Regulation of the blood-biliary barrier: interaction between gap and tight junctions in hepatocytes. Med. Electron Microsc. 36, 157–164. 10.1007/s00795-003-0220-5 14505059

[B34] Lewis C. T. A. Melhedegaard E. G. Ognjanovic M. M. Olsen M. S. Laitila J. Seaborne R. A. E. (2024). Remodelling of skeletal muscle myosin metabolic states in hibernating mammals. bioRxiv, 566992. 10.1101/2023.11.14.566992 PMC11098559 38752835

[B35] Malatesta M. Costanzo M. Cisterna B. Zancanaro C. (2020). Satellite cells in skeletal muscle of the hibernating dormouse, a natural model of quiescence and re-activation: focus on the cell nucleus. Cells 9, 1050. 10.3390/cells9041050 32340154 PMC7226265

[B36] Malatesta M. Perdoni F. Battistelli S. Muller S. Zancanaro C. (2009). The cell nuclei of skeletal muscle cells are transcriptionally active in hibernating edible dormice. BMC Cell. Biol. 10, 19. 10.1186/1471-2121-10-19 19284674 PMC2663540

[B37] Malatesta M. Zancanaro C. Baldelli B. Gazzanelli G. (2002). Quantitative ultrastructural changes of hepatocyte constituents in euthermic, hibernating and arousing dormice (muscardinus avellanarius). Tissue Cell. 34, 397–405. 10.1016/s0040816602000745 12441092

[B38] Miyazaki M. Shimozuru M. Kitaoka Y. Takahashi K. Tsubota T. (2022). Regulation of protein and oxidative energy metabolism are down-regulated in the skeletal muscles of asiatic black bears during hibernation. Sci. Rep. 12, 19723. 10.1038/s41598-022-24251-0 36385156 PMC9668988

[B39] Morrison S. F. Cao W. H. (2000). Different adrenal sympathetic preganglionic neurons regulate epinephrine and norepinephrine secretion. Am. J. Physiol. Regul. Integr. Comp. Physiol. 279, R1763–R1775. 10.1152/ajpregu.2000.279.5.R1763 11049860

[B40] Morrison S. F. Nakamura K. (2019). Central mechanisms for thermoregulation. Annu. Rev. Physiol. 81, 285–308. 10.1146/annurev-physiol-020518-114546 30256726

[B41] Morrison S. F. Sved A. F. Passerin A. M. (1999). Gaba-mediated inhibition of raphe pallidus neurons regulates sympathetic outflow to brown adipose tissue. Am. J. Physiol. 276, R290–R297. 10.1152/ajpregu.1999.276.2.R290 9950904

[B42] Nakamura K. Matsumura K. Hubschle T. Nakamura Y. Hioki H. Fujiyama F. (2004). Identification of sympathetic premotor neurons in medullary raphe regions mediating fever and other thermoregulatory functions. J. Neurosci. 24, 5370–5380. 10.1523/JNEUROSCI.1219-04.2004 15190110 PMC6729310

[B43] Nakamura K. Matsumura K. Kaneko T. Kobayashi S. Katoh H. Negishi M. (2002). The rostral raphe pallidus nucleus mediates pyrogenic transmission from the preoptic area. J. Neurosci. 22, 4600–4610. 10.1523/JNEUROSCI.22-11-04600.2002 12040067 PMC6758794

[B44] Nguyen N. L. Randall J. Banfield B. W. Bartness T. J. (2014). Central sympathetic innervations to visceral and subcutaneous white adipose tissue. Am. J. Physiol. Regul. Integr. Comp. Physiol. 306, R375–R386. 10.1152/ajpregu.00552.2013 24452544 PMC3949107

[B45] Paxinos G. Watson C. 2007. The rat brain in stereotaxic coordinates, p. Pages. Elsevier, San Diego. 10.1016/0165-0270(80)90021-7 6110810

[B46] Puspitasari A. Cerri M. Takahashi A. Yoshida Y. Hanamura K. Tinganelli W. (2021). Hibernation as a tool for radiation protection in space exploration. Life (Basel) 11, 54. 10.3390/life11010054 33466717 PMC7828799

[B47] Puspitasari A. Squarcio F. Quartieri M. Totis C. Hitrec T. Takahashi A. (2022). Synthetic torpor protects rats from exposure to accelerated heavy ions. Sci. Rep. 12, 16405. 10.1038/s41598-022-20382-6 36180516 PMC9525701

[B48] Reznik-Schuller H. Reznik G. (1974). The influence of hibernation upon the ultrastructure of the leydig cells and spermatids of the european hamster. Fertil. Steril. 25, 621–635. 10.1016/s0015-0282(16)40520-0 4842759

[B49] Ruf T. Bieber C. (2023). Why hibernate? Predator avoidance in the edible dormouse. Mamm. Res. 68, 1–11. 10.1007/s13364-022-00652-4 36624745 PMC9816287

[B50] Sgarbi G. Hitrec T. Amici R. Baracca A. Di Cristoforo A. Liuzzi F. (2022). Mitochondrial respiration in rats during hypothermia resulting from central drug administration. J. Comp. Physiol. B 192, 349–360. 10.1007/s00360-021-01421-6 35001173

[B51] Sone M. Yamaguchi Y. (2024). Cold resistance of mammalian hibernators approximately a matter of ferroptosis? Front. Physiol. 15, 1377986. 10.3389/fphys.2024.1377986 38725569 PMC11079186

[B52] Sonntag M. Arendt T. (2019). Neuronal activity in the hibernating brain. Front. Neuroanat. 13, 71. 10.3389/fnana.2019.00071 31338028 PMC6629779

[B53] Soria-Milla M. A. Coca-Garcia M. C. (1986). Ultrastructure of the proximal convoluted tubule in the hibernating garden dormouse (eliomys quercinus l.). Cryobiology 23, 537–542. 10.1016/0011-2240(86)90064-7 3802892

[B54] Standish A. Enquist L. W. Escardo J. A. Schwaber J. S. (1995). Central neuronal circuit innervating the rat heart defined by transneuronal transport of pseudorabies virus. J. Neurosci. 15, 1998–2012. 10.1523/JNEUROSCI.15-03-01998.1995 7891147 PMC6578173

[B55] Stenvinkel P. Jani A. H. Johnson R. J. (2013). Hibernating bears (ursidae): metabolic magicians of definite interest for the nephrologist. Kidney Int. 83, 207–212. 10.1038/ki.2012.396 23254895

[B56] Streefland C. Maes F. W. Bohus B. (1998). Autonomic brainstem projections to the pancreas: a retrograde transneuronal viral tracing study in the rat. J. Auton. Nerv. Syst. 74, 71–81. 10.1016/s0165-1838(98)00047-2 9915620

[B57] Takahashi T. M. Sunagawa G. A. Soya S. Abe M. Sakurai K. Ishikawa K. (2020). A discrete neuronal circuit induces a hibernation-like state in rodents. Nature 583, 109–114. 10.1038/s41586-020-2163-6 32528181

[B58] Takeichi M. (2014). Dynamic contacts: rearranging adherens junctions to drive epithelial remodelling. Nat. Rev. Mol. Cell. Biol. 15, 397–410. 10.1038/nrm3802 24824068

[B59] Ter Horst G. J. Van den Brink A. Homminga S. A. Hautvast R. W. Rakhorst G. Mettenleiter T. C. (1993). Transneuronal viral labelling of rat heart left ventricle controlling pathways. Neuroreport 4, 1307–1310. 10.1097/00001756-199309150-00005 8260610

[B60] Tupone D. Madden C. J. Morrison S. F. (2013). Central activation of the a1 adenosine receptor (a1ar) induces a hypothermic, torpor-like state in the rat. J. Neurosci. 33, 14512–14525. 10.1523/JNEUROSCI.1980-13.2013 24005302 PMC3761054

[B61] Wang Z. Jiang S. F. Cao J. Liu K. Xu S. H. Arfat Y. (2019). Novel findings on ultrastructural protection of skeletal muscle fibers during hibernation of daurian ground squirrels: mitochondria, nuclei, cytoskeleton, glycogen. J. Cell. Physiol. 234, 13318–13331. 10.1002/jcp.28008 30633347 PMC13483305

[B62] Yang Y. Yuan J. Field R. L. Ye D. Hu Z. Xu K. (2023). Induction of a torpor-like hypothermic and hypometabolic state in rodents by ultrasound. Nat. Metab. 5, 789–803. 10.1038/s42255-023-00804-z 37231250 PMC10229429

[B63] Zakharova N. M. Tarahovsky Y. S. Fadeeva I. S. Komelina N. P. Khrenov M. O. Glushkova O. V. (2019). A pharmacological composition for induction of a reversible torpor-like state and hypothermia in rats. Life Sci. 219, 190–198. 10.1016/j.lfs.2019.01.023 30658098

[B64] Zancanaro C. Malatesta M. Mannello F. Vogel P. Fakan S. (1999). The kidney during hibernation and arousal from hibernation. A natural model of organ preservation during cold ischaemia and reperfusion. Nephrol. Dial. Transpl. 14, 1982–1990. 10.1093/ndt/14.8.1982 10462281

[B65] Zancanaro C. Malatesta M. Merigo F. Benati D. Fakan S. Gazzanelli G. (2000). Berlin, Heidelberg.

[B66] Zaretsky D. V. Zaretskaia M. V. DiMicco J. A. (2003). Stimulation and blockade of gaba(a) receptors in the raphe pallidus: effects on body temperature, heart rate, and blood pressure in conscious rats. Am. J. Physiol. Regul. Integr. Comp. Physiol. 285, R110–R116. 10.1152/ajpregu.00016.2003 12609814

[B67] Zimny M. L. Levy E. D. Jr. (1971). Ultrastructure of mesangial and juxtaglomerular cells in the kidney of a hibernator. Z Zellforsch Mikrosk Anat. 118, 326–332. 10.1007/BF00331191 4327754

